# *catena*-Poly[(*S*)-2-methyl­piperazine-1,4-diium [[trichloridobismuthate(III)]-di-μ-chlorido]]

**DOI:** 10.1107/S1600536810031417

**Published:** 2010-08-11

**Authors:** Zong-Ling Ru

**Affiliations:** a Department of Chemical and Environmental Engineering, Anyang Institute of Technology, Anyang 455000, People’s Republic of China

## Abstract

In the crystal structure of the title compound, {(C_5_H_14_N_2_)[BiCl_5_]}_*n*_, the Bi^III^ cation is coordinated by six Cl^−^ anions in a distorted octa­hedral geometry. Two Cl^−^ anions bridge neighboring Bi^III^ cations, forming a zigzag polymeric chain along the *a* axis. The discrete methylpiperazinediium cation adopts a normal chair conformation and is linked to the polymeric chains by N—H⋯Cl hydrogen bonding.

## Related literature

For transition-metal complexes of 2-methyl­piperazine, see: Ye *et al.* (2009 ▶).
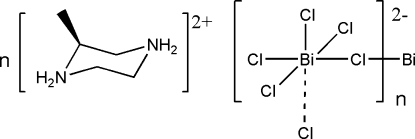


## Experimental

### 

#### Crystal data

(C_5_H_14_N_2_)[BiCl_5_]
*M* *_r_* = 488.41
Orthorhombic, 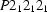

*a* = 7.719 (1) Å
*b* = 10.8997 (16) Å
*c* = 16.302 (3) Å
*V* = 1371.6 (3) Å^3^
*Z* = 4
Mo *K*α radiation
μ = 13.79 mm^−1^
*T* = 293 K
0.28 × 0.26 × 0.24 mm

#### Data collection

Rigaku SCXmini diffractometer
Absorption correction: multi-scan (*CrystalClear*; Rigaku, 2005 ▶) *T*_min_ = 0.8, *T*_max_ = 0.9
14082 measured reflections
3150 independent reflections
3009 reflections with *I* > 2σ(*I*)
*R*_int_ = 0.089

#### Refinement

*R*[*F* ^2^ > 2σ(*F* ^2^)] = 0.031
*wR*(*F* ^2^) = 0.066
*S* = 1.03
3150 reflections
120 parameters
H-atom parameters constrained
Δρ_max_ = 1.57 e Å^−3^
Δρ_min_ = −1.63 e Å^−3^
Absolute structure: Flack (1983 ▶), 1327 Friedel pairs
Flack parameter: −0.021 (9)

### 

Data collection: *CrystalClear* (Rigaku, 2005 ▶); cell refinement: *CrystalClear*; data reduction: *CrystalClear*; program(s) used to solve structure: *SHELXS97* (Sheldrick, 2008 ▶); program(s) used to refine structure: *SHELXL97* (Sheldrick, 2008 ▶); molecular graphics: *SHELXTL* (Sheldrick, 2008 ▶); software used to prepare material for publication: *SHELXL97*.

## Supplementary Material

Crystal structure: contains datablocks I, global. DOI: 10.1107/S1600536810031417/xu5013sup1.cif

Structure factors: contains datablocks I. DOI: 10.1107/S1600536810031417/xu5013Isup2.hkl

Additional supplementary materials:  crystallographic information; 3D view; checkCIF report

## Figures and Tables

**Table 1:** Selected bond lengths (Å)

Bi1—Cl1	2.8245 (18)
Bi1—Cl2	2.597 (2)
Bi1—Cl3	2.561 (2)
Bi1—Cl4	2.6135 (18)
Bi1—Cl5	2.875 (2)
Bi1—Cl5^i^	2.820 (2)

Symmetry code: (i) 
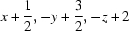
.

**Table 2:** Hydrogen-bond geometry (Å, °)

*D*—H⋯*A*	*D*—H	H⋯*A*	*D*⋯*A*	*D*—H⋯*A*
N1—H6*A*⋯Cl1^ii^	0.97	2.30	3.262 (7)	171
N1—H6*B*⋯Cl2	0.97	2.48	3.255 (7)	137
N1—H6*B*⋯Cl3	0.97	2.61	3.244 (6)	124
N2—H7*A*⋯Cl4^iii^	0.97	2.33	3.242 (7)	156
N2—H7*B*⋯Cl1^iv^	0.97	2.25	3.184 (6)	161

Symmetry codes: (ii) 
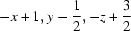
; (iii) 
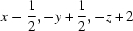
; (iv) 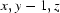.

**Fig. 1.**
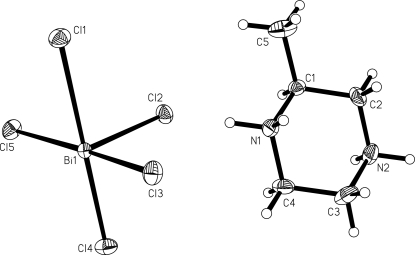
The asymmetric unit of the title compound with atom labels. Displacement ellipsoids were drawn at the 30% probability level.

**Fig. 2.**
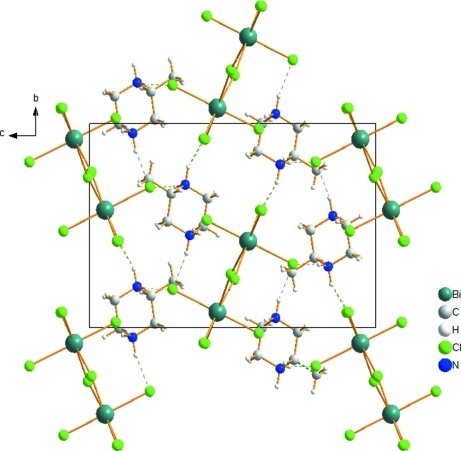
The packing viewed along the a axis. Hydrogen bonds are drawn as dashed lines

## supplementary crystallographic information

### Comment

The chiral 2-methylpiperazine has shown tremendous scope in the synthesis of transition metal complexes (Ye *et al.*, 2009). The construction of new members of this family of ligands is an important direction in the development of coordination chemistry. we report here the crystal structure of the title compound.

In the crystal of the title compound, C_5_H_14_N_2_.BiCl_5_ (Fig.1), the Bi^3+^ cations are coordinated by six Cl^- ^anions with distances ranging from 2.561 (2) to 2.875 (2) Å (Table 1). The values of bond angles Cl–Bi–Cl are near to 90 or 180°, which make the [BiCl_6_]^3-^ octahedral geometry. The protonated piperazine ring adopts a chair conformation. The Bi^3+^ cations conneted through bridging chlorine atom to form a one-dimensional chain structure. The crystal structure is stabilized by intermolecular N—H···Cl hydrogen bonds (Table 2).

### Experimental

A mixture of (*S*)-2-methylpiperazine (2 mmol, 0.2 g), BiCl_3_ (2 mmol, 0.62 g) and 20% aqueous HCl (20 ml) in 10 ml water was heated at 353 K for 0.5 h. The reaction mixture was cooled slowly to room temperature, crystals of the title compound were formed after 8 d.

### Refinement

All H atoms were placed in calculated positions, with C—H = 0.96 or 0.98 Å and N—H = 0.97 Å, and refined using a riding model, with *U*_iso_(H) = 1.5*U*_eq_(C) for methyl H atoms and 1.2*U*_eq_(C,*N*) for the others.

### Crystal data

**Table:**

(C_5_H_14_N_2_)[BiCl_5_]	*F*(000) = 904
*M**_r_* = 488.41	*D*_x_ = 2.365 Mg m^−^^3^
Orthorhombic, *P*2_1_2_1_2_1_	Mo *K*α radiation, λ = 0.71073 Å
Hall symbol: P 2ac 2ab	Cell parameters from 3009 reflections
*a* = 7.719 (1) Å	θ = 2.5–27.5°
*b* = 10.8997 (16) Å	µ = 13.79 mm^−^^1^
*c* = 16.302 (3) Å	*T* = 293 K
*V* = 1371.6 (3) Å^3^	Block, colorless
*Z* = 4	0.28 × 0.26 × 0.24 mm

### Data collection

**Table:**

Rigaku SCXmini diffractometer	3150 independent reflections
Radiation source: fine-focus sealed tube	3009 reflections with *I* > 2σ(*I*)
graphite	*R*_int_ = 0.089
Detector resolution: 13.6612 pixels mm^-1^	θ_max_ = 27.5°, θ_min_ = 2.5°
ω scans	*h* = −9→10
Absorption correction: multi-scan (*CrystalClear*; Rigaku, 2005)	*k* = −14→14
*T*_min_ = 0.8, *T*_max_ = 0.9	*l* = −21→21
14082 measured reflections	

### Refinement

**Table:**

Refinement on *F*^2^	Hydrogen site location: inferred from neighbouring sites
Least-squares matrix: full	H-atom parameters constrained
*R*[*F*^2^ > 2σ(*F*^2^)] = 0.031	*w* = 1/[σ^2^(*F*_o_^2^) + (0.*P*)^2^] where *P* = (*F*_o_^2^ + 2*F*_c_^2^)/3
*wR*(*F*^2^) = 0.066	(Δ/σ)_max_ = 0.001
*S* = 1.03	Δρ_max_ = 1.57 e Å^−^^3^
3150 reflections	Δρ_min_ = −1.63 e Å^−^^3^
120 parameters	Extinction correction: *SHELXL97* (Sheldrick, 2008), Fc^*^=kFc[1+0.001xFc^2^λ^3^/sin(2θ)]^-1/4^
0 restraints	Extinction coefficient: 0.0300 (5)
Primary atom site location: structure-invariant direct methods	Absolute structure: Flack (1983), 1327 Friedel pairs
Secondary atom site location: difference Fourier map	Flack parameter: −0.021 (9)

### Special details

**Table:**

Geometry. All e.s.d.'s (except the e.s.d. in the dihedral angle between two l.s. planes) are estimated using the full covariance matrix. The cell e.s.d.'s are taken into account individually in the estimation of e.s.d.'s in distances, angles and torsion angles; correlations between e.s.d.'s in cell parameters are only used when they are defined by crystal symmetry. An approximate (isotropic) treatment of cell e.s.d.'s is used for estimating e.s.d.'s involving l.s. planes.
Refinement. Refinement of *F*^2^ against ALL reflections. The weighted *R*-factor *wR* and goodness of fit *S* are based on *F*^2^, conventional *R*-factors *R* are based on *F*, with *F* set to zero for negative *F*^2^. The threshold expression of *F*^2^ > σ(*F*^2^) is used only for calculating *R*-factors(gt) *etc*. and is not relevant to the choice of reflections for refinement. *R*-factors based on *F*^2^ are statistically about twice as large as those based on *F*, and *R*- factors based on ALL data will be even larger.

### Fractional atomic coordinates and isotropic or equivalent isotropic displacement parameters (Å^2^)

**Table:**

	*x*	*y*	*z*	*U*_iso_*/*U*_eq_	
Bi1	0.33206 (3)	0.57633 (2)	0.946205 (14)	0.02412 (11)	
C1	0.1786 (10)	0.1571 (6)	0.7856 (4)	0.0296 (15)	
H1	0.0737	0.1733	0.8177	0.036*	
C2	0.1931 (12)	0.0212 (7)	0.7715 (5)	0.0391 (19)	
H2A	0.0899	−0.0077	0.7436	0.047*	
H2B	0.2917	0.0049	0.7362	0.047*	
C3	0.3672 (10)	−0.0026 (8)	0.8968 (6)	0.049 (2)	
H3A	0.4720	−0.0201	0.8661	0.058*	
H3B	0.3740	−0.0456	0.9487	0.058*	
C4	0.3548 (12)	0.1343 (8)	0.9124 (4)	0.041 (2)	
H4A	0.2566	0.1512	0.9477	0.050*	
H4B	0.4589	0.1625	0.9399	0.050*	
C5	0.1685 (13)	0.2283 (9)	0.7064 (5)	0.059 (2)	
H5A	0.1539	0.3139	0.7183	0.089*	
H5B	0.0717	0.1997	0.6748	0.089*	
H5C	0.2734	0.2166	0.6758	0.089*	
Cl1	0.3347 (3)	0.68787 (17)	0.78982 (11)	0.0414 (4)	
Cl2	0.0920 (2)	0.4253 (2)	0.89851 (12)	0.0378 (4)	
Cl3	0.5685 (2)	0.4239 (2)	0.90419 (13)	0.0389 (4)	
Cl4	0.3272 (3)	0.47345 (19)	1.09100 (11)	0.0412 (4)	
Cl5	0.0888 (3)	0.7630 (2)	0.99374 (14)	0.0414 (5)	
N1	0.3338 (8)	0.2005 (5)	0.8340 (4)	0.0331 (13)	
H6A	0.4376	0.1895	0.8012	0.040*	
H6B	0.3211	0.2875	0.8449	0.040*	
N2	0.2143 (8)	−0.0475 (5)	0.8499 (4)	0.0387 (16)	
H7A	0.1108	−0.0363	0.8828	0.046*	
H7B	0.2270	−0.1345	0.8388	0.046*	

### Atomic displacement parameters (Å^2^)

**Table:**

	*U*^11^	*U*^22^	*U*^33^	*U*^12^	*U*^13^	*U*^23^
Bi1	0.02674 (15)	0.02120 (14)	0.02443 (15)	−0.00003 (11)	−0.00047 (11)	−0.00161 (10)
C1	0.033 (3)	0.030 (4)	0.026 (3)	0.001 (3)	0.002 (3)	0.005 (3)
C2	0.054 (5)	0.032 (4)	0.031 (4)	−0.013 (4)	−0.001 (4)	−0.007 (3)
C3	0.045 (5)	0.040 (5)	0.060 (5)	−0.004 (4)	−0.017 (4)	0.021 (4)
C4	0.051 (5)	0.045 (5)	0.028 (4)	−0.013 (4)	−0.011 (4)	0.011 (3)
C5	0.062 (5)	0.076 (7)	0.040 (5)	0.004 (7)	0.000 (5)	0.023 (5)
Cl1	0.0513 (10)	0.0414 (10)	0.0316 (9)	0.0075 (11)	0.0079 (10)	0.0036 (8)
Cl2	0.0344 (9)	0.0359 (10)	0.0432 (10)	−0.0050 (9)	−0.0056 (8)	−0.0058 (10)
Cl3	0.0326 (8)	0.0350 (10)	0.0490 (11)	0.0048 (9)	0.0058 (8)	−0.0045 (11)
Cl4	0.0382 (9)	0.0521 (11)	0.0333 (9)	−0.0029 (11)	−0.0032 (9)	0.0123 (8)
Cl5	0.0440 (9)	0.0356 (11)	0.0446 (11)	0.0132 (8)	0.0096 (8)	0.0003 (9)
N1	0.043 (3)	0.026 (3)	0.031 (3)	−0.005 (3)	0.000 (3)	0.002 (2)
N2	0.039 (3)	0.024 (3)	0.053 (4)	−0.002 (3)	−0.003 (3)	0.001 (3)

### Geometric parameters (Å, °)

**Table:**

Bi1—Cl1	2.8245 (18)	C3—C4	1.517 (12)
Bi1—Cl2	2.597 (2)	C3—H3A	0.9700
Bi1—Cl3	2.561 (2)	C3—H3B	0.9700
Bi1—Cl4	2.6135 (18)	C4—N1	1.476 (9)
Bi1—Cl5	2.875 (2)	C4—H4A	0.9700
Bi1—Cl5^i^	2.820 (2)	C4—H4B	0.9700
C1—C2	1.504 (10)	C5—H5A	0.9600
C1—C5	1.509 (10)	C5—H5B	0.9600
C1—N1	1.510 (10)	C5—H5C	0.9600
C1—H1	0.9800	N1—H6A	0.9700
C2—N2	1.490 (10)	N1—H6B	0.9700
C2—H2A	0.9700	N2—H7A	0.9700
C2—H2B	0.9700	N2—H7B	0.9700
C3—N2	1.489 (10)		
			
Cl3—Bi1—Cl2	90.97 (6)	C4—C3—H3A	109.4
Cl3—Bi1—Cl4	88.48 (7)	N2—C3—H3B	109.4
Cl2—Bi1—Cl4	89.32 (7)	C4—C3—H3B	109.4
Cl3—Bi1—Cl5^i^	89.71 (8)	H3A—C3—H3B	108.0
Cl2—Bi1—Cl5^i^	177.10 (7)	N1—C4—C3	110.0 (6)
Cl4—Bi1—Cl5^i^	87.88 (7)	N1—C4—H4A	109.7
Cl3—Bi1—Cl1	91.88 (7)	C3—C4—H4A	109.7
Cl2—Bi1—Cl1	90.44 (7)	N1—C4—H4B	109.7
Cl4—Bi1—Cl1	179.57 (7)	C3—C4—H4B	109.7
Cl5^i^—Bi1—Cl1	92.35 (7)	H4A—C4—H4B	108.2
Cl3—Bi1—Cl5	175.20 (8)	C1—C5—H5A	109.5
Cl2—Bi1—Cl5	93.63 (8)	C1—C5—H5B	109.5
Cl4—Bi1—Cl5	92.91 (7)	H5A—C5—H5B	109.5
Cl5^i^—Bi1—Cl5	85.753 (13)	C1—C5—H5C	109.5
Cl1—Bi1—Cl5	86.75 (6)	H5A—C5—H5C	109.5
C2—C1—C5	112.3 (7)	H5B—C5—H5C	109.5
C2—C1—N1	109.2 (7)	Bi1^ii^—Cl5—Bi1	172.74 (9)
C5—C1—N1	109.0 (6)	C4—N1—C1	112.7 (6)
C2—C1—H1	108.7	C4—N1—H6A	109.0
C5—C1—H1	108.7	C1—N1—H6A	109.2
N1—C1—H1	108.7	C4—N1—H6B	109.3
N2—C2—C1	111.8 (6)	C1—N1—H6B	108.8
N2—C2—H2A	109.3	H6A—N1—H6B	107.8
C1—C2—H2A	109.3	C3—N2—C2	111.2 (6)
N2—C2—H2B	109.3	C3—N2—H7A	109.1
C1—C2—H2B	109.3	C2—N2—H7A	108.7
H2A—C2—H2B	107.9	C3—N2—H7B	109.7
N2—C3—C4	111.1 (7)	C2—N2—H7B	110.0
N2—C3—H3A	109.4	H7A—N2—H7B	108.0

Symmetry codes: (i) *x*+1/2, −*y*+3/2, −*z*+2; (ii) *x*−1/2, −*y*+3/2, −*z*+2.

### Hydrogen-bond geometry (Å, °)

**Table:**

*D*—H···*A*	*D*—H	H···*A*	*D*···*A*	*D*—H···*A*
N1—H6A···Cl1^iii^	0.97	2.30	3.262 (7)	171.
N1—H6B···Cl2	0.97	2.48	3.255 (7)	137.
N1—H6B···Cl3	0.97	2.61	3.244 (6)	124.
N2—H7A···Cl4^iv^	0.97	2.33	3.242 (7)	156.
N2—H7B···Cl1^v^	0.97	2.25	3.184 (6)	161.

Symmetry codes: (iii) −*x*+1, *y*−1/2, −*z*+3/2; (iv) *x*−1/2, −*y*+1/2, −*z*+2; (v) *x*, *y*−1, *z*.
